# Simultaneous Determination of Hydrochlorothiazide and Losartan Potassium in Pharmaceutical Product by UV-Vis Spectrophotometric Method with Kalman Filter Algorithm

**DOI:** 10.1155/2021/2754133

**Published:** 2021-07-20

**Authors:** Tran Thuc Binh, Le Thi Phuong Tram, Nguyen Van Hop, Nguyen Dang Giang Chau, Nguyen Duy Luu, Nguyen Thi Quynh Trang

**Affiliations:** ^1^University of Sciences, Hue University, Hue 530000, Vietnam; ^2^Da Nang University of Medical Technology and Pharmacy, Da Nang 550000, Vietnam; ^3^Faculty of Environmental Sciences, Saigon University, Ho Chi Minh City 700000, Vietnam

## Abstract

In this paper, hydrochlorothiazide (HCT) and losartan potassium (LSP) in tablets are simultaneously determined with UV-Vis spectrophotometry and chemometrics without separation. The spectra of standard and sample solutions were recorded in the wavelength from 220 to 300 nm at 1.0 nm intervals. The concentrations of HCT and LSP in the sample solutions were computed with the Kalman filter algorithm written on the Microsoft Excel 2016 and Visual Basic for Applications (VBA) platform. The method validation was determined via the accuracy and repeatability of measurements when analyzing HCT and LSP in the Splozarsin Plus tablet and comparing the mean values of their contents in the sample with those analyzed with HPLC. The proposed method is simple with a low cost compared with the standard HPLC method.

## 1. Introduction

Molecular absorption spectrometry incorporating chemometrics is often used to determine components in a mixture with overlapping spectra simultaneously. The general principle of this combined method is that the full-spectral data are loaded into software programmed with algorithms and statistical methods, from which each component in the mixture is quantified. Due to its simplicity, the method is considered environmentally friendly and economical. Besides, full-spectral data for calculations combined with algorithms help to increase the accuracy of the determination. Standard chemometrics methods include classical least squares (CLS), partial least squares (PLS), Kalman filter, principal component regression (PCR), artificial neural networks (ANNs), and derivative spectrophotometry. These methods have been studied and applied to various matrices [1–7].

In our previous study [4], we succeeded in employing the Kalman filter algorithm with full-spectral measurement to build a program (written on the Microsoft Excel 2016 and Visual Basic for Applications (VBA) platform) for determining the concentration of substances in a mixture. Inheriting these results, we continue modifying and applying this combined method (from now on, Kalman-Excel method) to determine Hydrochlorothiazide (HCT) and Losartan Potassium (LSP) in Splozarsin Plus simultaneously. Hydrochlorothiazide (C_7_H_8_ClN_3_O_4_S_2_, *M* = 297.7 g/mol) is a diuretic medicine (or water pills) used to treat hydronephrosis, renal failure, or nephropathy. In addition, HCT is also used effectively to prevent and treat hypertension, liver disorders, myocardial infarction, heart failure, or stroke in patients with cardiovascular problems. Losartan Potassium (C_22_H_22_ClKN_6_O, *M* = 461.0 g/mol) belongs to a class of medicine called angiotensin receptor blockers that dilate blood vessels, facilitating blood to flow more easily. Losartan Potassium is used to treat high blood pressure and heart failure and help to protect the kidney damage caused by diabetes [8–10].

For the determination of HCT and LSP in medicine, various techniques have been developed elsewhere. Maggio et al. [8] established a procedure based on the multivariate analysis of spectral data in the 220–274 nm region, with the partial least square algorithm. The results agreed well with those determined with HPLC. Rathee et al. [9] developed Vierordt's and Q absorbance ratio methods. They were successfully applied to the assay of LSP and HCT in tablet formulations. Rao et al. [10] built three techniques for this purpose: (1) to solve the equations simultaneously; (2) to determine the absorbance ratio at 272 nm (the maximum absorption of HCT) and 266.5 nm (the isosbestic wavelength); (3) to use the first-order derivative method. Thube et al. [11] also proposed three spectrophotometric methods for the simultaneous determination of LSP and HCT in a binary mixture: (1) to measure the absorbance at 235 nm and 271 nm, corresponding to the absorbance maxima of LSP and HCT, respectively (the concentration of each drug was obtained by using the absorption values calculated for both drugs at these wavelengths); (2) to use the dual-wavelength principle in which the absorbance difference between two points on the mixture spectra was directly proportional to the concentration of the component of interest and independent of the interfering components; and (3) to describe and calculate the area under the curve between 265 and 282 nm for LSP and between 229 and 242 nm for HCT. The proposed methods were applied to marketed formulation. High-performance liquid chromatography was also applied to quantify these two compounds in pharmaceutical products [12–15]. Although HPLC delivers simultaneous determination, high cost prevents its application.

As the absorption spectra of HCT and LSP are overlapped, the Kalman filter algorithm using full spectra (Kalman-Excel method) [4] is employed in this study to quantify these two substances in pharmaceutical products simultaneously. The proposed method opens up the possibility of a fast and cheap technique for pharmaceutical analysis and testing.

## 2. Materials and Methods

### 2.1. Instrument and Chemicals

#### 2.1.1. Instruments

A UV-Vis spectrophotometer Cary 60 (Agilent, USA) for spectral scanning (190–990 nm) is connected to a computer with Cary WinUV software for spectral data storage (in Excel spreadsheets). All the measurements were carried out at the Laboratory of Applied Chemistry, Department of Chemistry, University of Sciences, Vietnam

#### 2.1.2. Chemicals

Hydrochlorothiazide standard (C_7_H_8_ClN_3_O_4_S_2_), 99.8% purity, was purchased from the National Institute of Drug Quality Control, standard Vietnamese pharmacopoeia (Code: C0219308.02). Losartan Potassium standard (C_22_H_22_ClKN_6_O), 99.9% purity, was obtained from the Institute of Drug Quality Control, Ho Chi Minh City, standard Vietnamese pharmacopoeia (Lot No. 166090520). All other chemicals used were of analytical grade.

The stock solution of HCT (50.0 *μ*g/mL) was prepared by dissolving 12.5 mg of HCT standard in 150 mL of distilled water and ultrasonicated for 15 min. The solution was then made up to 250 mL with distilled water. Twenty-five millilitres of the stock solution was diluted with 75 mL of distilled water to make a working solution of HCT (12.5 *μ*g/mL). The stock solution of LSP (50.0 *μ*g/mL) (also acted as a working solution) was prepared by dissolving 12.5 mg LSP standard in distilled water and making up to 250 mL.

### 2.2. Sample Preparation

In this study, Splozarsin Plus tablets (Co. Ltd. SHINPOONG DAEWOO, Bien Hoa industrial zone, Dong Nai, Vietnam, batch code 9003, manufactured date 5 June 2019, expired date 5 June 2021) were used for the method validation. The reference amount of LSP and HCT on the label is 50.0 mg and 12.5 mg, respectively.

Twenty tablets with an average weight of 253.4 mg were ground to a fine powder and mixed well. A particular amount of the powder (*m*) was dissolved in 150 mL distilled water and ultrasonicated for 30 min before filtering to make up a 250 mL solution. Ten millilitres of the solution was diluted to 100 mL with distilled water for measurements.

The amount of each target compound in a tablet is expressed via mg/tablet and calculated according to the following equation:

(1) $$\begin{array}{c} \frac{\text{mg}}{\text{tablet}}=\;C_{i}\times 100\times \left(\frac{250}{10}\right)\times \left(\frac{M}{m}\right)\times \left(\frac{1}{1000}\right)=2.5\times C_{i}\times \left(\frac{M}{m}\right)\text{,} \end{array}$$

where *C*_*i*_ (*μ*g/mL) is the concentration of compound *i* in the final solution; *M* (mg) is the average weight of the tablets; and *m* (mg) is the weight of the powder.

### 2.3. Analytical Procedure

To determine HCT and LSP simultaneously, we utilized the Kalman filter algorithm with full-spectral measurements. The Visual Basic for Applications, a programming language written on the basis of Microsoft Excel 2016, was used for data treatment [4].

Briefly, the standard solutions of individual compounds and their actual sample solutions were subjected to record molecular absorption spectra (scanned from 220 to 300 nm wavelength at 1.0 nm intervals). The achieved data were saved as .csv files and extracted to the computer as Excel sheets. The developed Kalman-Excel program was applied to determine the concentrations and respective errors.

### 2.4. Method Validation

#### 2.4.1. Relative Error (RE%)

The relative error (RE%) between concentrations is calculated according to the following equation [16]:

(2) $$\begin{array}{c} \text{RE}\left(\text{\%}\right)=\frac{\left(C-C_{0}\right)}{100}\times \;C_{0}, \end{array}$$

where *C* is the measured concentration (*μ*g/mL) and *C*_0_ is the concentration of the standard solution (*μ*g/mL).

#### 2.4.2. Limit of Detection (LOD) and Limit of Quantification (LOQ)

Measurements are made on a minimum of seven aliquots (*n* ≥ 7) of a prepared sample solution that has a concentration near the expected limit of detection [16]. The developed Kalman-Excel program was applied to determine the concentrations of HCT and LSP in the seven aliquots when using their spectrum data and spectrum data of the standard solutions of HCT and LSP. The LOD and LOQ were then calculated by the following formula:

(3) $$\begin{array}{c} \text{LOD}=3\text{.}3\times \text{SD,}\\ \text{LOQ}=10\times \text{SD,} \end{array}$$

where SD is the standard deviation of seven replicates.

#### 2.4.3. Repeatability (RSD%)

The repeatability of the analytical method is assessed via relative standard deviation [16–18]:

(4) $$\begin{array}{c} \text{RSD}\left(\%\right)=\frac{\text{SD}\times 100}{C_{m}}, \end{array}$$

where SD is the standard deviation and *C*_m_ is the mean concentration of *n* replicates.

The RSD value is acceptable if lower than 1/2RSD_Horwitz_ [19], in which RSD_Horwitz_ is calculated according to the following equation:

(5) $$\begin{array}{c} {\text{RSD}}_{\text{Horwitz}}=2^{\left(1-0.5\;\times \;\mathrm{lg}C\right)}, \end{array}$$

where *C* is expressed as a fraction (e.g., *C* = 5 *μ*g/mL = 5 × 10^−6^).

#### 2.4.4. Trueness

The trueness of the analytical method was verified via the method's recovery and the mean values of the developed method and a standard method.

*(i) Recovery*. The mean recovery is determined by assessing spiked samples at three different levels (three replicates per level) of each target compound, namely, 0.500, 1.000, and 1.500 *μ*g/mL for HCT and 2.000, 4.000, and 6.000 *μ*g/mL for LSP. The recovery rate is calculated by the following equation:

(6) $$\begin{array}{c} \text{Rev}\left(\%\right)=\;\frac{\left(C{}_{2}-C{}_{1}\right)\times 100}{C_{s}}, \end{array}$$

where *C*_2_ (*μ*g/mL) is the measured concentration of the spiked sample; *C*_1_ (*μ*g/mL) is the measured concentration of the original sample (sample without spiking); and *C*_s_ (*μ*g/mL) is the spiked concentration.

In this study, Splozarsin Plus tablets were used as the original sample. Three-fifths of the average tablet weight (3/5 × *M*) were taken for each sample. The standard solutions of HCT (5.000 *μ*g/mL) and LSP (20.000 *μ*g/mL) were also prepared at the same time for spectral measurements. The developed Kalman-Excel program was applied to quantify the concentration of HCT and LSP in the samples simultaneously. The acceptable recovery rate is from 85% to 110% [18].

*(ii) Comparing Two Sample Means Performed By Kalman-Excel Method and Standard HPLC Method*. According to Harvey [20], the trueness of an analytical method could be evaluated by analyzing *n*_1_ and *n*_2_ replicates with the developed method and the standard method. The mean values achieved from the two analytical methods are compared with the *t*-test. The Splozarsin Plus tablets were sent to the Drug, Cosmetic and Food Quality control Center of Thua Thien Hue Province for analysis with the HPLC method. Hydrochlorothiazide and Losartan Potassium were quantified following the Vietnam Pharmacopoeia V guideline [20]. The Splozarsin Plus solutions were prepared (described in Section 2.2) and analyzed with the developed Kalman-Excel method (described in Section 2.3) with three replicates. The *t*-test was used for evaluation: when the variances for the two analyses are not significantly different if the experimental *t* value is lower than the *t* value at risk of 0.05 and (*n*_1_ + *n*_2−2_) degrees of freedom, the mean values achieved from the two methods are not significantly different.

## 3. Results and Discussion

### 3.1. Relative Errors and Repeatability

**Figure 1:**
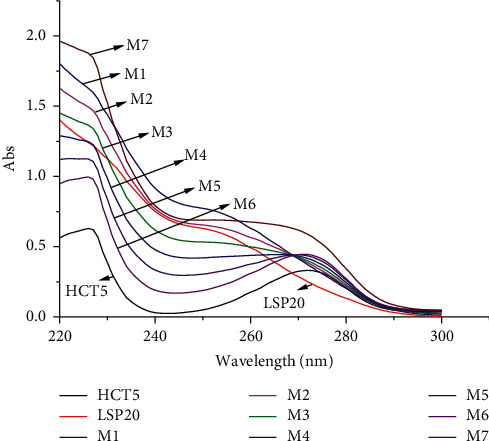
Absorption spectra of the HCT and LSP standards and their mixtures at different concentration ratios. HCT5: HCT standard solution of 5.000 *μ*g/mL; LSP20: LSP standard solution of 20.000 *μ*g/mL; M1: mixture of HCT 1.000 *μ*g/mL + LSP 24.000 *μ*g/mL; M2 : mixture of HCT 2.000 *μ*g/mL + LSP 20.000 *μ*g/mL; M3: mixture of HCT 3.000 *μ*g/mL + LSP 16.000 *μ*g/mL; M4: mixture of HCT 4.000 *μ*g/mL + LSP 12.000 *μ*g/mL; M5: mixture of HCT 5.000 *μ*g/mL + LSP 8.000 *μ*g/mL; M6: mixture of HCT 6.000 *μ*g/mL + LSP 4.000 *μ*g/mL; M7: mixture of HCT 5.000 *μ*g/mL + LSP 20.000 *μ*g/mL.

Seven mixtures of HCT and LSP standards with different concentration ratios were prepared from the standard solutions of HCT (12.500 *μ*g/mL) and LSP (50.000 *μ*g/mL). The mixtures and the standard solutions were subjected to analysis with the Kalman-Excel method, as described in Section 2.3. The absorption spectra are shown in Figure 1.

At different HCT and LSP concentration ratios, the RE values between reference concentrations and their measured concentrations vary from −1.55 to 1.93% (Table 1). In addition, the RSD values are far lower than the respective 1/2RSD_H_ values. This finding indicates the good performance of the Kalman-Excel method for the simultaneous determination of HCT and LSP in standard mixtures.

### 3.2. Method Validation on Pharmaceutical Tablets

#### 3.2.1. Quantification of HCT and LSP in Splozarsin Plus Tablet and Method Repeatability

**Figure 2:**
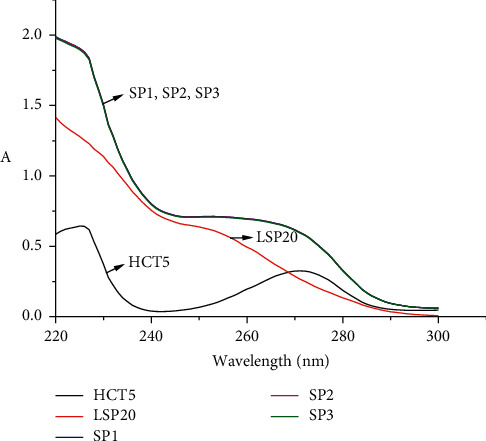
Absorption spectra of HCT and LSP standard solutions and Splozarsin Plus sample solutions. HCT5: standard solution of HCT 5.000 *μ*g/mL; LSP20: standard solution of LSP 20.000 *μ*g/mL; SP1, SP2, SP3: Splozarsin Plus sample solutions.

A Splozarsin Plus powder weighting 253.4 mg (after grinding and mixing) was prepared as described in Section 2.2 and analyzed as described in Section 2.3. The amount of HCT and LSP in the Splozarsin Plus tablet was determined following equation (1). The absorption spectra of the sample and standard solutions are illustrated in Figure 2.

The results in Table 2 show that the Kalman-Excel method to determine HCT and LSP in a pharmaceutical product simultaneously yields good repeatability (RSDs vary from 0.27 to 0.3% for two studied compounds). The average amounts of HCT and LSP quantified in Splozarsin Plus tablet are 12.42 **±** 0.09 mg and 50.02 **±** 0.34 mg, respectively. These values agree well with the reference content on the label (12.5 mg and 50 mg, respectively). They also comply with the quality standards of the Vietnamese Ministry of Health, which allows the content of the ingredients in this type of tablets as follows: HCT: 12.50 mg ± 7.5% (11.56–13.44 mg), LSP: 50.00 mg ± 5% (47.5–52.5 mg) [21].

#### 3.2.2. Trueness, LOD, and LOQ of Method

**Figure 3:**
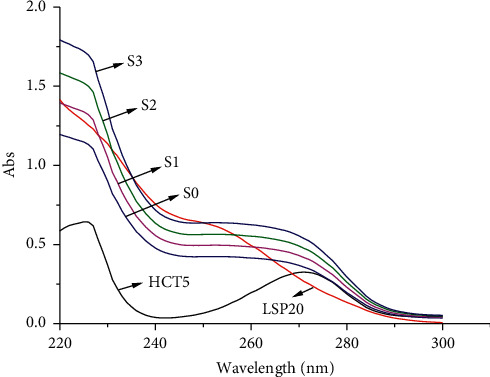
Absorption spectra of standard solutions, spiked samples, and original sample. HCT5: standard solution of HCT 5 *μ*g/mL; LSP20: standard solution of LSP 20 *μ*g/mL; S0: original samples; S1: spiked sample level 1 (HCTad = 0.500 *μ*g/mL, LSPad = 2.000 *μ*g/mL); S2: spiked sample level 2 (HCTad = 1.000 *μ*g/mL, LSPad = 4.000 *μ*g/mL); S3: spiked sample level 3 (HCTad = 1.500 *μ*g/mL, LSPad = 6.000 *μ*g/mL).

*(1) Recovery of the Method*. The absorption spectra of standard solutions and spiked samples are demonstrated in Figure 3. The results of recovery are shown in Table 3.

The recovery of the Kalman-Excel method is approximately 100% for the studied compounds with good repeatability (RSD% < 0.167, *n* = 3). This result is comparable with that of the previous studies [8–14], which employed different spectrophotometry methods for simultaneous determination of HCT and LSP in tablets (Table 4).

In addition to that, the LOD and LOQ values achieved in this method were 0.037 and 0.12 *μ*g/mL for HCT and 0.228 and 0.76 *μ*g/mL for LSP (Table 4). Accordingly, it was an advance over the results from the studies of Rao et al. [10] who used three different spectrophotometry methods including simultaneous equation, ratio of absorbance, and first-order derivative method to quantify HCT and LSP in tablets, or the studies of Mohammed et al. [13] and Srinivasu et al. [14] who applied RP-HPLC (Table 4).

Besides the good performance of the developed method, another highlight was its simplicity in sample preparation, in which H_2_O was the only solvent used for sample dilution and preparation, while most of the other methods consumed various organic solvents (Table 4).

(2) *Comparing Two Sample Means Performed by Kalman-Excel Method and HPLC Method*. To continue evaluating the method trueness, we compare the concentration of HCT and LSP in the Splozarsin Plus tablets quantified with the Kalman-Excel method with that of the HPLC method. The *t*-test is used for data comparison.

The data in Table 5 demonstrate that *t*_exp_ is lower than the *t* (0.05; 4), implying that the analytical results of the two methods are statistically consistent with the significance level of 0.05. In other words, the Kalman-Excel method exhibits good trueness. In addition, the *F*_exp_ values are lower than the *F* (0.05, 2, 2) when *α* = 0.05, revealing that the developed analytical method and the standard method (HPLC) produce the same exact repeatability.

## 4. Conclusions

We successfully apply full-spectral measurements with the modified Kalman filter algorithm method for the simultaneous determination of Hydrochlorothiazide and Losartan Potassium in pharmaceutical formulation tablets. The method minimizes the time needed for sample preparation as well as the amounts of solvent used and the waste generated. The analytical procedure exhibits good repeatability, good trueness, and low relative errors for the studied compounds. There is no statistically significant difference in analytical results between the Kalman-Excel method and the standard HPLC procedure used as reference. The simplicity and good sample throughput of this method make it suitable for routine quality control testing of the pharmaceutical products containing simultaneously Hydrochlorothiazide and Losartan Potassium.

## Figures and Tables

**Table 1:** Relative errors and repeatability of the Kalman-Excel method.

Sample name	Conc. ratios (HCT/LSP)	*n*	HCT			LSP		
			*C*_HCT_ (*μ*g/mL)	RE (%)	RSD (1/2RSD_H_) (%)	*C*_LSP_ (*μ*g/mL)	RE (%)	RSD (1/2RSD_H_) (%)
M1	1 : 24	1	1.002	0.20	0.30	24.206	0.86	0.07
		2	0.996	−0.40	(8.00)	24.238	0.99	(4.96)
		3	0.999	−0.10		24.229	0.95	
		Average	0.990	−0.10		24.224	0.93	

M2	2 : 20	1	1.981	−0.95	0.175	20.115	0.57	0.05
		2	1.981	−0.95	(7.21)	20.112	0.56	(5.10)
		3	1.975	−1.25		20.131	0.66	
		Average	1.979	−1.05		20.119	0.6	

M3	3 : 16	1	2.982	−0.60	0.166	15.961	−0.24	0.06
		2	2.973	−0.90	(6.78)	15.971	−0.18	(5.27)
		3	2.974	−0.87		15.979	−0.13	
		Average	2.976	−0.79		15.970	−0.18	

M4	4 : 12	1	3.975	−0.62	0.14	12.067	0.56	0.02
		2	3.966	−0.85	(6.49)	12.071	0.59	(5.50)
		3	3.965	−0.87		12.072	0.60	
		Average	3.969	−0.78		12.070	0.58	

M5	5 : 08	1	5.008	0.16	0.20	8.019	0.24	0.10
		2	4.991	−0.18	(6.28)	8.035	0.44	(5.85)
		3	4.990	−0.20		8.031	0.39	
		Average	4.996	−0.07		8.028	0.36	

M6	6 : 04	1	5.921	−1.32	0.21	4.064	1.60	0.17
		2	5.897	−1.72	(6.11)	4.074	1.85	(6.49)
		3	5.904	−1.60		4.077	1.93	
		Average	5.907	−1.55		4.072	1.79	

M7	5 : 20	1	4.990	−0.20	0.15	20.203	1.02	0.06
		2	4.975	−0.50	(6.28)	20.224	1.12	(5.10)
		3	4.980	−0.40		20.220	1.10	
		Average	4.982	−0.37		20.216	1.08	

RSD_H_: RSD value calculated from the Horwitz function; M1: mixture of HCT 1.000 *μ*g/mL + LSP 24.000 *μ*g/mL; M2: mixture of HCT 2.000 *μ*g/mL + LSP 20.000 *μ*g/mL; M3: mixture of HCT 3.000 *μ*g/mL + LSP 16.000 *μ*g/mL; M4: mixture of HCT 4.000 *μ*g/mL + LSP 12.000 *μ*g/mL; M5: mixture of HCT 5.000 *μ*g/mL + LSP 8.000 *μ*g/mL; M6: mixture of HCT 6.000 *μ*g/mL + LSP 4.000 *μ*g/mL; M7: mixture of HCT 5.000 *μ*g/mL + LSP 20.000 *μ*g/mL.

**Table 2:** Measured amounts of HCT and LSP in Splozarsin Plus tablet and the respective RSD values.

Sample name	HCT^*∗*^		LSP^*∗*^	
	*C*_HCT_ (*μ*g/mL)	Content (mg/tablet)	CLSP (*μ*g/mL)	Content (mg/tablet)
SP1	4.978	12.45	20.033	50.08
SP2	4.975	12.44	20.042	50.11
SP3	4.953	12.38	19.942	49.86
Average	4.969	12.42	20.01	50.02
RSD%	0.29	0.30	0.27	0.27

^*∗*^The amounts of LSP and HCT on the Splozarsin Plus label were 50.0 mg and 12.5 mg, respectively.

**Table 3:** Recovery of HCT and LSP in Splozarsin Plus.

Sample	HCT				LSP			
	*C*_spiked_ (*μ*g/mL)	*C*_measured_ (*μ*g/mL)	Rev (%)	RSD (%)	*C*_spiked_ (*μ*g/mL)	*C*_measured_ (*μ*g/mL)	Rev (%)	RSD (%)
S_01_	0	3.003	—	0.167	0	11.997	—	0.029
S_02_		3.009	—		0	11.991	—	
S_03_		3.013	—		0	11.997	—	

S_11_	0.500	3.512	101.80	0.066	2.000	13.962	98.25	0.014
S_12_		3.508	99.80		2.000	13.964	98.65	
S_13_		3.508	99.00		2.000	13.966	99.45	

S_21_	1.000	3.996	99.30	0.100	4.000	15.887	97.25	0.017
S_22_		4.004	99.50		4.000	15.882	97.28	
S_23_		4.000	98.70		4.000	15.886	97.73	

S_31_	1.500	4.515	100.80	0.056	6.000	17.973	99.60	0.010
S_32_		4.520	100.73		6.000	17.973	99.73	
S_33_		4.517	100.27		6.000	17.970	99.88	

**Table 4:** The validation results of other methods in comparison with this Kalman-Excel method for the determination of HCT and LSP in tablets.

Sources	Method	Solvent	Range of concentration (*μ*g/mL) HCT LSP	LOD (*μ*g/mL) HCT LSP	LOQ (*μ*g/mL) HCT LSP	Rev (%) HCT LSP	RSD (%) HCT LSP
[8]	PLS, spectrophotometry	H_2_O	1.06–5.70 4.0–22.2	0.06 0.21	0.14 0.49	95.6 93.5	0.6 1.7
[9]	1/Vierordt's method 2/absorbance ratio method	0.01N HCl	0.563–27.27 0.708–22.22	0.233 0.708	0.186 0.563	98.13–99.69	<0.246
[10]	1/simultaneous equation 2/ratio of absorbance 3/first-order derivative method	Methanol	1/1–20 5–25 2/1–25 5–80 3/1–40 1–30	0.5	1.97	≈99	0.57
[11]	1/simultaneous equation 2/dual wavelength 3/area under curve		5–30	0.30 0.30 (3 methods)	0.90 0.90 (3 methods)	98.7–100.3	0.49–0.57 0.49–0.57 (3 methods)
[12]	Microemulsion liquid chromatography	Mobile phase SDS, n-butanol, n-octane, water, and acetonitrile	2.5–12.5 10.0–60.0	0.03 0.04	0.1 0.15	98.9 101	<1.4
[13]	RP-HPLC	Acetonitrile and formic acid	2–10 5–50	0.5 for HCT and LSP	1.5 for HCT and LSP	96 92	<0.61 <1.22
[14]	RP-HPLC	Acetonitrile and formic acid	1.0–300.3 4.4–240.2	0.304 0.232	0.921 0.734	99.55-100.67 98.44-100.48	0.24–0.61 0.19–1.22
This study	Spectrophotometry with Kalman filter algorithm	H_2_O	1.0–6.0 4.0–24.0	0.037 0.228	0.12 0.76	98.7–101.8 97.25–99.88	<0.167 for HCT and LSP

**Table 5:** Performance of Kalman-Excel method versus HPLC method to quantify HCT and LSP in Splozarsin Plus tablet simultaneously (*n*_1_ = *n*_2_ = 3).

Amount of HCT in a tablet (mg/tablet)		Amount of LSP in a tablet (mg/tablet)	
Kalman-Excel	HPLC	Kalman-Excel	HPLC
12.4450	12.45	50.0825	49.93
12.4375	12.38	50.105	50.30
12.3825	12.42	49.855	50.45
Mean = 12.422 ± 0.034	Mean = 12.417 ± 0.035	Mean = 50.014 ± 0.138	Mean = 50.227 ± 0.268
*F*_exp_ = 1.059		*F*_exp_ = 3.745	
*F* (0.05; 2; 2) = 39.00		*F* (0.05; 2; 2) = 39.00	
SD_pool_ = 0.0346		SD_pool_ = 0.2130	
*t*_exp_ = 0.177		*t*_exp_ = 1.222	
*t* (0.05, 4) = 2.776		*t* (0.05, 4) = 2.776	

*F*_exp_: experimental variance of the method; *F* (0.05, 2, 2): *F* distribution at alpha 0.05 and the respective degrees of freedom of numerator and denominator; S_pool_: pooled variance; *t*_exp_: experimental *t* value, *t* (0.05, 4): *t* value at risk of 0.05 and 4 degrees of freedom.

## Data Availability

The data used to support the finding of this study are available from the corresponding author upon request.
